# Psychiatric Admissions, Referrals, and Suicidal Behavior Before and During the COVID-19 Pandemic in Denmark: A Time-Trend Study

**DOI:** 10.1192/j.eurpsy.2022.1359

**Published:** 2022-09-01

**Authors:** T. Rømer, R. Christensen, S. Blomberg, F. Folke, H. Christensen, M. Benros

**Affiliations:** 1 University Hospital Copenhagen, Copenhagen Research Center For Mental Health (core), Hellerup, Denmark; 2 University of Copenhagen, Copenhagen Emergency Medical Services, Ballerup, Denmark

**Keywords:** Admissions, Referrals, Suicide, Covid-19

## Abstract

**Introduction:**

The COVID-19 pandemic has affected mental health globally, but the impact on referrals and admissions to mental health services remains understudied.

**Objectives:**

To assess patterns in psychiatric admissions, referrals, and suicidal behavior before and during the COVID-19 pandemic in Denmark.

**Methods:**

Utilizing hospital and Emergency Medical Services (EMS) health records covering 46% of the Danish population, we compared psychiatric in-patients, referrals to mental health services and suicidal behavior in years prior to the COVID-19 pandemic to levels during the first lockdown (March 11 – May 17, 2020), inter-lockdown period (May 18 – December 15, 2020), and second lockdown (December 16, 2020 – February 28, 2021) using negative binomial models.

**Results:**

The rate of psychiatric in-patients declined compared to pre-pandemic levels (RR = 0.95, 95% CI = 0.94 – 0.96, p < 0.01). Referrals were not significantly different (RR = 1.01, 95% CI = 0.92 – 1.10, p = 0.91) during the pandemic; neither was suicidal behavior among hospital contacts (RR = 1.04, 95% CI = 0.94 – 1.14, p = 0.48) nor EMS contacts (RR = 1.08, 95% CI = 1.00 – 1.18, p = 0.06). In the age group <18, an increase in the rate of psychiatric in-patients (RR = 1.11, 95% CI = 1.07 – 1.15, p < 0.01) was observed during the pandemic; however, this did not exceed the pre-pandemic, upwards trend in psychiatric hospitalizations in the age group <18 (p = 0.78).

**Conclusions:**

The pandemic was associated with a decrease in psychiatric hospitalizations. No significant change was observed in referrals and suicidal behavior.

**Disclosure:**

No significant relationships.

